# Gut microbiota-derived metabolites as context-dependent modulators of Nrf2 signaling in inflammation-driven carcinogenesis

**DOI:** 10.1080/19490976.2026.2718561

**Published:** 2026-08-20

**Authors:** My-Lan Pianka, Xuelei Zhang, Christoph K. Stein-Thoeringer

**Affiliations:** a Department of Infectious Diseases, Medical Microbiology and Hygiene, Medical Faculty Heidelberg, Heidelberg University, Heidelberg, Germany; b University Hospital Heidelberg, Heidelberg, Germany; c M3 Research Center, Faculty of Medicine, University of Tübingen, Tübingen, Germany; d Department of Internal Medicine I, Faculty of Medicine, University of Tübingen, Tübingen, Germany

**Keywords:** Microbial metabolites, Nrf2, redox homeostasis, chronic inflammation, inflammation-associated cancer, clinical translation

## Abstract

Chronic inflammation fosters cancer development by sustaining redox imbalance, oxidative stress and persistent tissue damage. A key component to this process is the transcription factor Nrf2 (nuclear factor erythroid 2-related factor 2), which coordinates antioxidant and detoxification programs to maintain redox homeostasis. While transient Nrf2 activation protects non-malignant tissues by limiting cell and tissue damage, persistent or dysregulated Nrf2 signaling in premalignant tissue and established tumors can promote cancer cell survival, metabolic adaptation and resistance to chemotherapy. Growing evidence identifies gut microbiota-derived metabolites as modulators of Nrf2 signaling, thereby providing a mechanistic link between microbial metabolism and inflammation-driven carcinogenesis. Distinct functional classes of bacterial metabolites, including short-chain fatty acids, tryptophan-derived indoles, secondary bile acids, polyphenol-derived metabolites and electrophilic redox-active compounds, converge on Nrf2 regulation across epithelial, immune and tumor compartments. Importantly, the biological consequences of these interactions are highly context-dependent and shaped by disease stage, cellular metabolic state and tissue microenvironment.

This review integrates current evidence on how microbial metabolites regulate Nrf2 signaling and redox homeostasis during chronic inflammation and cancer development or progression. We further discuss how dietary interventions, probiotics or postbiotics, and metabolite-based strategies may be leveraged to modulate the microbiota-Nrf2 axis for cancer prevention or therapy. Overall, we propose that microbial metabolites represent a context-dependent mechanism for modulating Nrf2 activity in inflammation-associated cancers, with important implications for translational and precision medicine approaches.

## 1. Introduction

Inflammation-driven cancers arise in chronically inflamed tissues, where persistent cytokine signaling and sustained production of reactive oxygen and nitrogen species (ROS/RNS) promote DNA damage, aberrant survival signaling and remodeling of a tumor-supportive microenvironment^1^ ^,^ ^2^ Central to the cellular response to such stress is the transcription factor Nrf2 (nuclear factor erythroid 2-related factor 2, encoded by NFE2L2), a master regulator of antioxidant response and associated detoxification and metabolic programs. Accumulating evidence indicates that Nrf2 signaling exerts context-dependent functions, with biological outcomes shaped by cellular and metabolic states and by disease stage. The same Nrf2-dependent transcriptional programs may be protective in premalignant tissues yet tumor-supportive once malignant transformation has occurred.^3^

Beyond host-intrinsic stress-response pathways, microbiota-derived metabolites have emerged as key modulators of redox and inflammatory signaling.^4^ ^,^ ^5^ Many of these metabolites directly or indirectly interact with Nrf2, either by imposing oxidative or electrophilic stress or by intersecting with upstream metabolic and receptor-mediated pathways. Through this metabolite-driven modulation of Nrf2 activity, gut microbes can regulate host stress adaptation across epithelial, immune and tumor compartments.

Importantly, the intestinal microbiome and its metabolite output are not static but undergo profound compositional and functional shifts across disease states. The transition from mucosal homeostasis to chronic inflammation and malignant transformation is accompanied by changes in microbial community structure and host metabolism,^6^ ^,^ ^7^ as well as alterations in the intestinal metabolite milieu.^8-11^ Consequently, distinct metabolites or metabolite classes may become more prominent across health, inflammatory disease or cancer, potentially influencing redox balance and immune activation.

This review focuses on gut microbiota-derived metabolites as context-dependent regulators of Nrf2 signaling in chronic inflammation and cancer. Rather than assigning uniform pro- or anti-tumor roles to individual metabolites or metabolite classes, we highlight how identical metabolite-Nrf2 interactions can yield divergent biological outcomes depending on tissue compartments, inflammatory states and stages of carcinogenesis. Understanding this context dependency is essential for rational translation of microbiota-Nrf2 interactions into cancer prevention or therapeutic strategies across the inflammation-cancer continuum.

## 2. Nrf2 signaling in carcinogenesis and tumor microenvironment

### 2.1. Dual role of Nrf2 in inflammation-driven carcinogenesis

Nrf2 is a central transcription factor for cellular redox homeostasis and stress adaptation, integrating oxidative, electrophilic and metabolic cues to coordinate antioxidant, detoxification and metabolic gene programs. Under physiological conditions, Nrf2 activity is tightly controlled by Keap1 (Kelch-like ECH-associated protein 1)-mediated ubiquitination and proteasomal degradation, enabling rapid and reversible pathway activation in response to cellular stress.^12^ Through this regulation, Nrf2 limits excessive reactive oxygen and nitrogen species accumulation, preserves cellular integrity and constrains inflammatory damage, thereby shaping tissue responses to inflammatory stress.

In the context of chronic inflammation, Nrf2 signaling plays a dual role in carcinogenesis.^13^ ^,^ ^14^ During early inflammatory stages, transient Nrf2 activation is generally tissue-protective, limiting oxidative DNA damage, supporting epithelial barrier integrity and dampening pro-inflammatory signaling. Genetic and experimental models consistently demonstrate that loss of Nrf2 exacerbates inflammation-driven tissue injury and increases susceptibility to carcinogenesis.^15^ Importantly, the transition from Nrf2-mediated tissue protection to tumor-supportive stress adaptation is unlikely to occur abruptly at tumor onset. Rather, accumulating evidence suggests that during late-stage chronic inflammation and premalignant disease, persistent or repetitive Nrf2 activation can promote stress-adapted cell states that may facilitate survival under chronic stress conditions.^16^ ^,^ ^17^ This may enable the persistence of damaged cells, thereby increasing the likelihood of malignant transformation.^3^ Following malignant transformation, such adaptive Nrf2 programs often become stabilized, frequently driven by Keap1 pathway alterations or non-canonical activation mechanisms,^17^ and can be exploited to support metabolic adaptation, stress tolerance and resistance to chemotherapy.^18^ ^,^ ^19^

**Figure 1.**
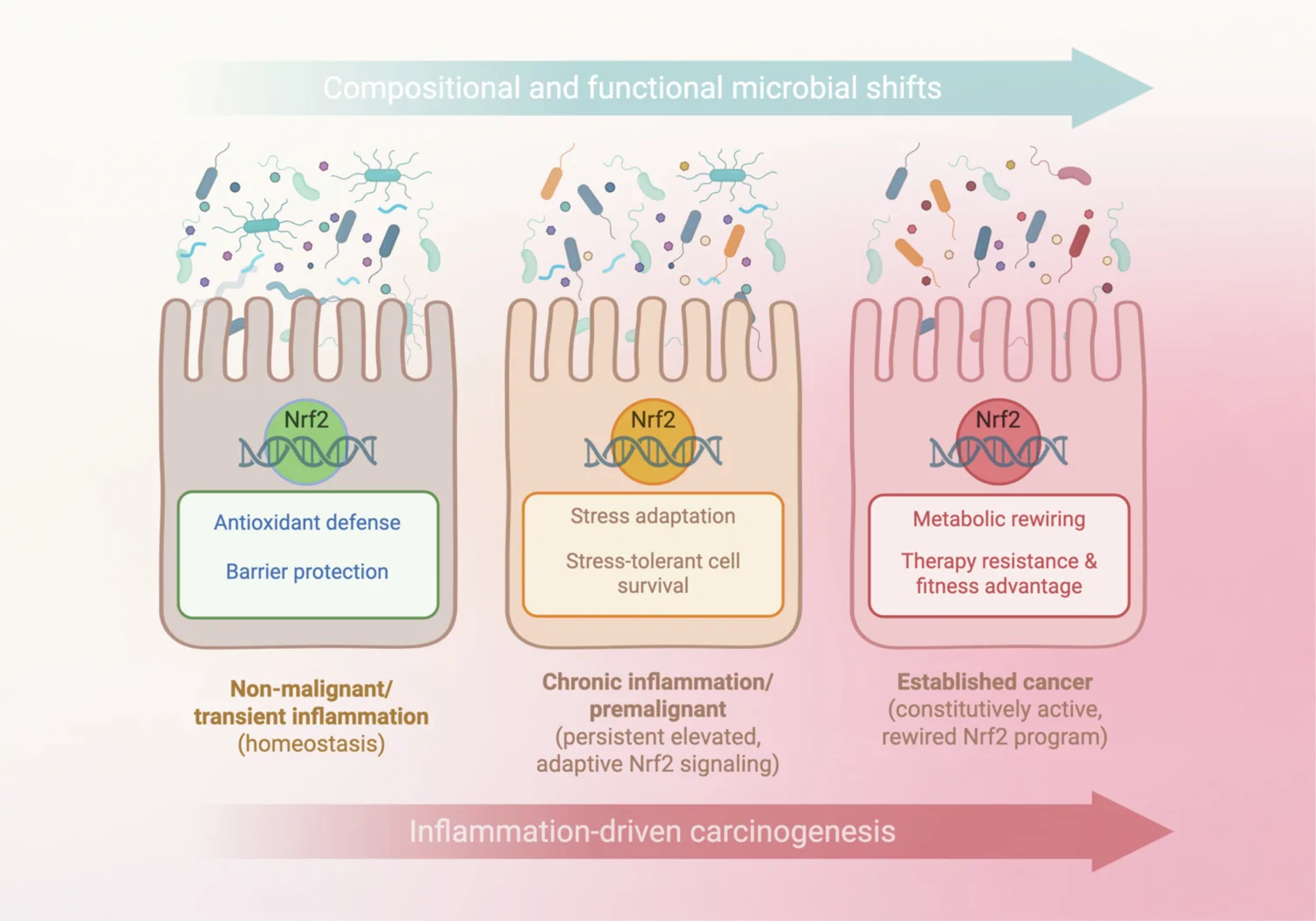
Disease stage-dependent microbial shifts and functional reprogramming of Nrf2 signaling. During progression from mucosal homeostasis to chronic inflammation and established cancer, the intestinal microbiota and its metabolite output undergo compositional and functional changes that are paralleled by stage-specific alterations in Nrf2 activity. In non-malignant conditions, a balanced metabolite milieu supports physiological Nrf2 signaling, contributing to antioxidant defense and epithelial barrier integrity. During chronic inflammation, altered microbial configurations reshape the metabolite landscape and promote persistently elevated, adaptive Nrf2 activation associated with stress-tolerant cell states. Following malignant transformation, tumor-associated microbial and metabolic changes further shape the metabolite milieu and contribute to constitutive Nrf2 activation and functional rewiring, supporting metabolic adaptation, stress tolerance and resistance to therapy. Created in BioRender. Pianka, M. (2026) https://BioRender.com/n6h661x.

Together, these observations indicate that Nrf2 does not conform to the classical definition of either a tumor suppressor or an oncogene. Rather, it operates as a context-dependent stress-adaptation module whose biological output is shaped by inflammatory burden, metabolic wiring and disease stage (Figure 1).

### 2.2. Cell type-specific roles of Nrf2 in the tumor microenvironment

Nrf2 exerts distinct functions within the tumor microenvironment, particularly across immune cell populations. In tumor-associated macrophages, Nrf2 activation has been linked to immunosuppressive and pro-metastatic programs, including heme-driven stress responses that promote tumor invasion, angiogenesis and resistance to immunotherapy.^20^ ^,^ ^21^ Similarly, Nrf2 regulates the survival and suppressive activity of myeloid-derived suppressor cells, thereby contributing to inhibition of T-cell function.^22^

In lymphoid compartments, Nrf2 modulates T-cell responses in a context-dependent manner. While excessive Nrf2 activation can impair antitumor immunity, targeted modulation of redox-sensitive Nrf2 signaling has been shown to enhance CD8⁺ T-cell and CAR-T-cell activity under conditions of oxidative stress.^23^ In addition, tumor-intrinsic activation of the KEAP1-Nrf2 pathway has been associated with immune evasion, including reduced dendritic-cell and T-cell activity and impaired responses to immune checkpoint blockade.^24^

Beyond immune cells, Nrf2 signaling also contributes to stromal remodeling. In cancer-associated fibroblasts, redox-dependent activation of Nrf2 promotes pro-survival and tumor-supportive programs, including enhanced stress tolerance and extracellular matrix remodeling.^25^ At the same time, Nrf2 activation in non-malignant stromal compartments can exert context-dependent restraining effects on tumor progression, highlighting the importance of compartment-specific signaling.^26^ Consistent with this, recent studies indicate that Nrf2-driven oxidative stress responses differ across stromal cell populations within the tumor microenvironment.^27^

These observations highlight the diverse, cell type-specific roles of Nrf2 within the tumor microenvironment, which is particularly relevant for understanding how microbiota-derived metabolites modulate Nrf2 signaling across epithelial and microenvironmental contexts.

## 3. Bacterial metabolites targeting Nrf2

Nrf2 is highly sensitive to metabolic and electrophilic cues,^28^ rendering it a prominent convergence point for gut microbiota-derived metabolites. Increasing evidence implicates these metabolites as modulators of Nrf2 signaling. Rather than exerting uniform effects, these metabolites interact with Nrf2 through diverse molecular routes and elicit distinct biological outcomes depending on tissue type, compartment and disease stage. This context dependency provides a conceptual framework for understanding how the same metabolite-Nrf2 interaction can shift from protective in non-malignant settings to tumor-supportive once malignancy is established.

In this section, we summarize major classes of microbial metabolites that influence Nrf2 activity (Table 1), with particular emphasis on intestinal milieus where microbial exposure is most pronounced and clinically relevant. Metabolites are discussed in functional classes to highlight shared mechanistic principles. Divergent cancer-related outcomes are interpreted primarily as context-dependent consequences of compartment- and stage-specific Nrf2 signaling rather than properties of individual metabolites. At the same time, certain metabolites may exert preferential effects in specific pathological settings.

**Table 1.** Microbiota-derived metabolites modulating Nrf2 signaling in context of carcinogenesis. The table summarizes representative metabolites within each class and their reported effects on Nrf2-related pathways. Effects are highly context-dependent and reflect the experimental systems investigated; absence of tumor-promoting effects does not imply tumor-suppressive activity. The level of evidence for Nrf2 involvement is classified as experimentally assessed Nrf2 pathway activation, inferred Nrf2 involvement based on ROS-/stress-associated responses without direct Nrf2 assessment, or indirect modulation through upstream signaling pathways. Metabolites were selected based on their microbiota-derived origin and reported relevance to cancer initiation, progression, or therapy response.

Metabolite class	Specific metabolite	Experimental context	Evidence for Nrf2 involvement	Biological outcome	Key reference(s)
SCFA	Butyrate	Non-malignant intestinal epithelium; DSS colitis (mouse)	Nrf2 activation (experimentally demonstrated; redox/mitophagy-associated)	Barrier protection, anti-inflammatory	[29]
		Human CRC cell lines (Warburg phenotype)	Inferred (redox/metabolic stress; Nrf2 not directly assessed)	Growth suppression, epigenetic reprogramming	[30–32]
Indoles	Indole-3-propionic acid (IPA)	Intestinal epithelium	Nrf2 activation (experimentally demonstrated; barrier-associated genes)	Enhanced epithelial barrier	[33]
		Breast cancer cell lines	Inferred (oxidative stress; Nrf2 not directly assessed)	Cytostatic effects; growth suppression	[34]
	Indole-3-lactic acid (ILA)	Intestinal epithelium; ischemia/reperfusion	Indirect (AhR-dependent signaling; Nrf2 not directly assessed)	Barrier and vascular protection	[35,36]
		Colorectal cancer models	Inferred (metabolic reprogramming; Nrf2 not directly assessed)	Tumor growth suppression	[37]
	Indole-3-aldehyde (IAld/I3A)	Immune and epithelial compartments	Indirect (AhR-associated signaling; Nrf2 not directly assessed)	Immune homeostasis; inflammation control	[38]
Urolithins	Urolithin A	Intestinal epithelium	Nrf2 activation (experimentally demonstrated)	Barrier protection	[39]
		Human supplementation study	Inferred (mitophagy-associated; Nrf2 not directly assessed)	Enhanced mitochondrial and cellular health	[40]
		CRC cell lines	Inferred (ROS-associated stress; Nrf2 not directly assessed)	Cell cycle arrest; apoptosis	[41–43]
Bile acids	Ursodeoxycholic acid	Hepatic and esophageal epithelial cells	Nrf2 activation (experimentally demonstrated)	Cytoprotection; antioxidant response	[44–46]
	Lithocholic acid	Cancer vs non-malignant cells	Inferred (ROS-associated stress; Nrf2 not directly assessed in tumor studies)	Cytotoxic or adaptive responses (context-dependent)	[47–49]
	Deoxycholic acid (DCA)	Barrett/epithelial cells	Nrf2 activation (experimentally demonstrated)	Protection against oxidative stress and DNA damage; enhanced cell survival	[50,51]
Polyamines	Spermidine	Colitis; liver injury models	Nrf2 activation (experimentally demonstrated; non-canonical mechanism)	Barrier protection; antifibrotic effects	[52,53]
		Cancer models	Inferred (stress adaptation; Nrf2 not directly assessed)	Therapy resistance (context-dependent)	[54,55]
Gasotransmitters	Hydrogen sulfide (H₂S)	Intestinal and vascular tissues	Nrf2 activation (experimentally demonstrated; KEAP1 S-sulfhydration)	Cytoprotection; adaptive stress response	[56,57]
	Nitric oxide (NO)	Colon carcinoma and epithelial cells	Nrf2 activation (experimentally demonstrated; KEAP1 S-nitrosylation)	Redox adaptation; survival signaling	[58,59]
Electrophiles	Acrolein	Epithelial cells	Nrf2 activation (experimentally demonstrated; electrophilic KEAP1 modification)	Redox stress response	[60–62]
	Reuterin	CRC models; epithelial cells	Inferred (electrophilic stress; Nrf2 not directly assessed)	Redox stress; tumor suppression	[63–65]
	Fatty acid derivatives, 10-oxo-octadecenoic acid (KetoC)	Epithelial and immune cells	Nrf2 activation (experimentally demonstrated; electrophilic stress-associated)	Anti-inflammatory; cytoprotection	[66–69]
Phenolics	3,4-Dihydroxyphenylacetic acid (DOPAC)	CRC models; immune cells	Nrf2 activation (experimentally demonstrated; mitophagy-associated)	Tumor suppression; immune activation	[70]

Given that Nrf2 activation is primarily mediated through redox- and electrophile-dependent modification of KEAP1 rather than classical ligand-receptor interactions, attribution of Nrf2 modulation to specific upstream signals remains challenging. In the context of this review, direct Nrf2 activation refers to mechanisms that engage the canonical KEAP1-Nrf2 regulatory axis, in which oxidative or electrophilic stress leads to chemical modification of key cysteine residues in KEAP1, resulting in stabilization, nuclear translocation, and transcriptional activation of Nrf2.

Accordingly, in Table 1, we distinguish between (i) experimentally demonstrated Nrf2 activation, (ii) conditions in which oxidative or electrophilic stress has been experimentally observed, suggesting Nrf2 pathway engagement in the absence of direct Nrf2 assessment, and (iii) indirect modulation via upstream signaling pathways such as AhR, which do not directly engage the KEAP1-Nrf2 axis but instead regulate cellular responses through parallel transcriptional programs.

Across microbial metabolite classes, the experimental context in which links between metabolite exposure and Nrf2 signaling have been established varies substantially. While some metabolites have been studied directly in chronically inflamed or tumor-bearing tissues, others are primarily characterized in non-malignant or homeostatic epithelial tissues and their relevance to carcinogenesis is inferred from effects on barrier integrity and modulation of inflammatory susceptibility.

### 3.1. Short-chain fatty acids (SCFAs)

Gut microbiota-derived short-chain fatty acids (SCFAs), particularly butyrate, emerge as relevant upstream modulators of Nrf2 signaling in the intestine. SCFAs are major end-products of microbial fiber fermentation and link diet, the microbiota and host inflammatory state.^4^ ^,^ ^71^ While acetate and propionate contribute substantially to the overall SCFA pool,^71^ butyrate has been most consistently associated with epithelial redox regulation and, in selected contexts, Nrf2-dependent cytoprotective responses.

In inflamed intestinal tissues, butyrate attenuates oxidative stress and inflammatory signaling, including suppression of NF-κB/NLRP3 activity and induction of antioxidant and mitochondrial pathways, accompanied by increased Nrf2 expression and activation of Nrf2-associated pathways.^29^ This represents one of the contexts in which Nrf2 activation has been experimentally demonstrated following butyrate exposure in inflamed intestinal models. However, beyond this setting, direct experimental assessment of Nrf2 activation remains comparatively limited.

**Figure 2.**
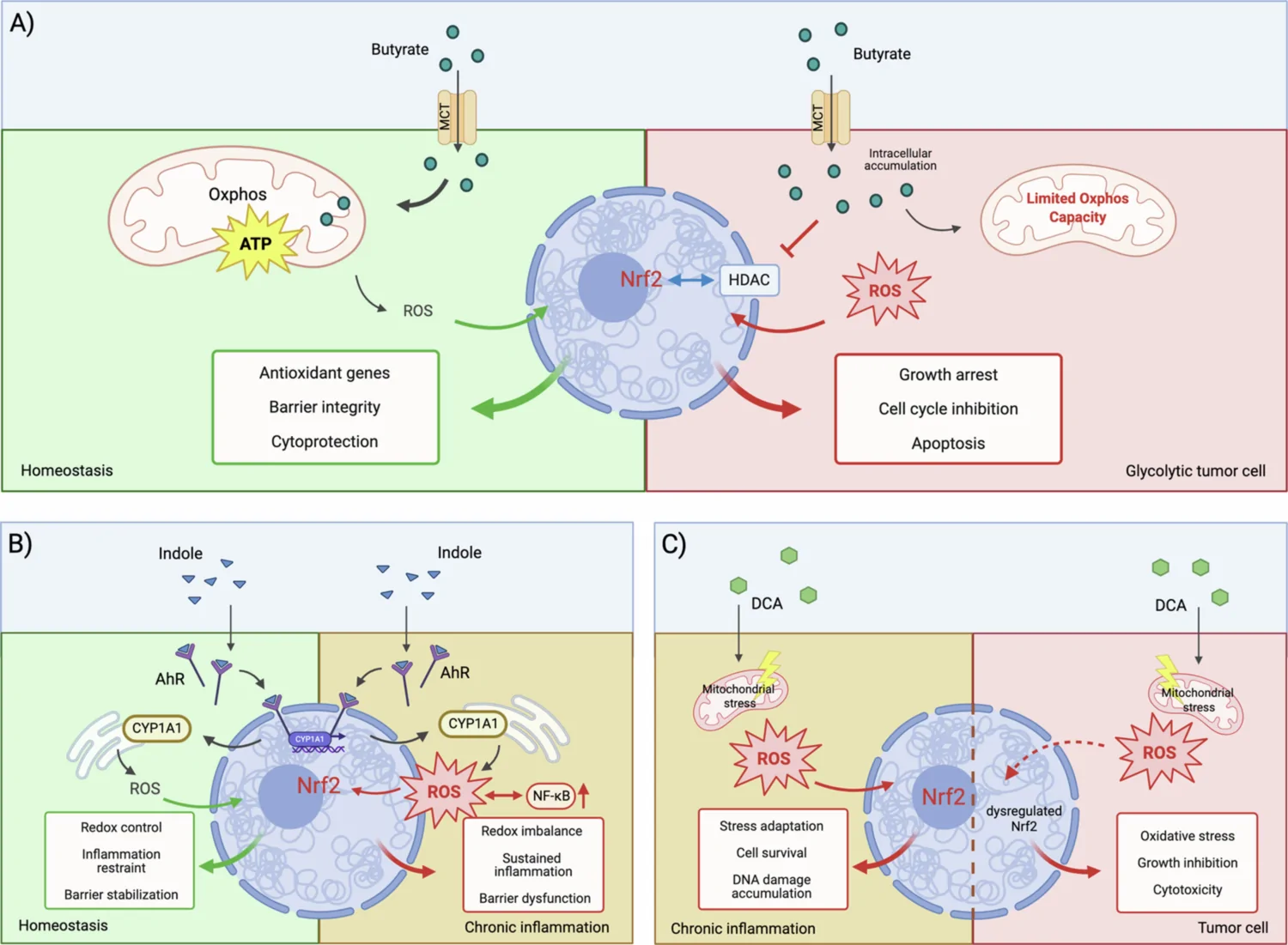
Context-dependent Nrf2 responses illustrated by selected microbiota-derived metabolites across metabolic, inflammatory and disease-stage conditions. Although these contexts are interconnected, each panel highlights a dominant axis shaping Nrf2 signaling outcomes. (A) Metabolic context. In non-transformed epithelial cells, butyrate is efficiently utilized through oxidative phosphorylation, supporting ATP production and maintaining redox balance. Under these conditions, Nrf2 activation promotes antioxidant gene expression, epithelial barrier integrity and cytoprotection. In contrast, in glycolytic tumor cells with limited oxidative capacity, intracellular butyrate accumulation enhances histone deacetylase (HDAC) inhibition and increases oxidative stress, resulting in growth arrest, cell cycle inhibition and apoptosis. (B) Inflammatory context. Indole derivatives signal through the intracellular aryl hydrocarbon receptor (AhR), which translocates to the nucleus and induces target genes such as CYP1A1, contributing to redox regulation and epithelial barrier homeostasis. During chronic inflammation, increased oxidative stress and persistent NF-κB activation alter the epithelial redox environment, shifting adaptive AhR/Nrf2-associated responses toward dysregulated stress signaling and impaired barrier function. (C) Disease-stage context. Secondary bile acids such as deoxycholic acid (DCA) induce mitochondrial stress and reactive oxygen species (ROS) production in epithelial cells. In chronically inflamed tissues, this promotes Nrf2-dependent stress adaptation, enabling cell survival despite ongoing DNA damage and contributing to inflammation-associated carcinogenesis. In contrast, in established tumor cells, the Nrf2 response may become impaired or insufficient depending on tumor context, resulting in increased oxidative stress, growth inhibition and cytotoxicity. Created in BioRender. Pianka, M. (2026) https://BioRender.com/kcc23t6.

In non-malignant epithelial cells, butyrate is predominantly utilized as an oxidative substrate that supports mitochondrial metabolism and contributes to redox homeostasis,^30^ conditions that are consistent with redox-dependent Nrf2 activation but do not demonstrate Nrf2 involvement. By contrast, in highly glycolytic colorectal cancer cells with limited oxidative capacity, intracellular accumulation of butyrate promotes HDAC inhibition^30^ and amplifies oxidative and metabolic stress responses (Figure 2). In this metabolic context, butyrate has been reported to suppress Nrf2 signaling, while also enhancing oxidative stress and inhibiting proliferation via growth arrest or apoptosis.^30-32^ ^,^ ^72^ Although direct comparisons across metabolically stratified tumor models remain limited, these findings support the concept that cellular metabolic state is a key determinant of butyrate sensitivity. Extending this concept to the human setting, studies examining SCFA levels or butyrate-producing taxa in colorectal cancer report heterogeneous associations, with several cohorts showing reduced butyrate-producing taxa or fecal butyrate levels in patients compared to cancer-free controls, while others report no significant differences or variable findings across populations.^9^ ^,^ ^73^ ^,^ ^74^ Together, these findings indicate that SCFAs, particularly butyrate, can modulate Nrf2-associated responses in a context-dependent manner. However, systematic investigation of Nrf2 signaling in response to SCFAs remains limited, and in many settings Nrf2 involvement is inferred from associated changes in oxidative stress or cellular metabolism rather than directly assessed.

### 3.2. Tryptophan-derived indoles

Tryptophan-derived indoles are gut microbiota-generated metabolites arising from bacterial tryptophan catabolism in the intestine, with production shaped by microbial and dietary inputs.^75^ ^,^ ^76^ These compounds modulate redox balance and inflammation primarily through aryl hydrocarbon receptor (AhR) signaling and, in selected tissue contexts, have been associated with Nrf2-dependent antioxidant programs, although in many cases this interaction is indirectly mediated via AhR signaling.^35^ ^,^ ^36^ ^,^ ^77-79^ Accordingly, the extent of evidence linking indole metabolites to Nrf2 signaling varies substantially across compounds, disease states and experimental systems.

In non-tumorigenic conditions, including homeostatic, barrier-stressed and inflammatory models, indole metabolites promote gut epithelial barrier integrity, enhance mucus and tight-junction programs and attenuate inflammatory signaling.^33^ ^,^ ^35^ ^,^ ^80^ Direct evidence linking these effects to Nrf2 signaling remains limited. One example is indole-3-propionic acid (IPA), which has been demonstrated to induce Nrf2 expression and activate downstream barrier-associated genes in intestinal epithelial models.^33^ Beyond their role in epithelial homeostasis, microbiota-derived indoles appear to exert context-dependent biological activities. IPA promotes barrier-associated programs in non-malignant intestinal tissues but induces cytostatic responses in experimental tumor models through AhR- and PXR-dependent signaling accompanied by modulation of oxidative stress pathways.^34^ Likewise, ILA has been linked to tissue-protective effects in intestinal injury models but suppresses colorectal tumor growth through metabolic reprogramming in malignant settings.^34^ ^,^ ^37^ While both oxidative stress responses and metabolic reprogramming can intersect with Nrf2-regulated networks, Nrf2 involvement was not directly assessed in either study.

In addition to direct tumor-suppressive effects, indole derivatives can also exert indirect antitumor activities through modulation of the tumor microenvironment, including effects on T-cell differentiation and responses to immune checkpoint blockade.^76^ For example, indole-3-aldehyde (I3A) has been shown to influence tumor immunogenicity and immune responses via AhR-dependent pathways.^38^

Overall, the current evidence suggests that AhR represents the primary mechanistic framework through which microbiota-derived indoles have been studied. In contrast, relatively few studies directly examine Nrf2 signaling, despite the frequent observation of biological effects that could plausibly involve Nrf2-regulated pathways.

### 3.3. Bile acid-derived metabolites

Secondary bile acids are generated by gut bacterial metabolism of host-derived primary bile acids^81^ ^,^ ^82^ and represent an important class of microbiota-derived metabolites influencing epithelial stress in the gastrointestinal tract. In chronic intestinal inflammation, altered bile acid pools can impose sustained oxidative and genotoxic stress on epithelial tissues.^83^ ^,^ ^84^ Such stress conditions have been associated with activation of Nrf2-dependent antioxidant pathways. As an example, ursodeoxycholic acid (UDCA) has been experimentally demonstrated to induce oxidative or electrophilic stress, which engages the canonical KEAP1-Nrf2 regulatory axis, leading to cytoprotective and detoxification responses. This is consistent with a protective response to bile acid-associated stress to limit oxidative DNA damage and enhancing cellular resilience.^44-46^ ^,^ ^85^ These mechanistic observations have provided a rationale for the use of UDCA as a preventive intervention in conditions characterized by chronic bile acid exposure. However, clinical studies in Barrett’s esophagus suggest that, despite favorable alterations in bile acid composition, UDCA does not consistently reduce epithelial markers of oxidative DNA damage or cell turnover, highlighting the limited evidence for durable cancer-preventive effects.^86^ This limited efficacy may, in part, reflect the complex composition of bile acid pools under chronic exposure conditions, where bile acid species with functionally opposing effects may coexist. In addition, such activation may not be sufficient to ensure long-term cytoprotection under sustained exposure conditions. As a result, epithelial cells may persist despite ongoing bile acid-induced damage, allowing stress-adapted or altered epithelial cells to accumulate over time, consistent with models of inflammation-associated carcinogenesis.^48^ ^,^ ^49^

In contrast to more hydrophilic bile acids, which are generally associated with adaptive cytoprotective responses, hydrophobic secondary bile acids such as deoxycholic acid (DCA) promote epithelial oxidative stress, DNA damage, and have been linked to increased genotoxicity.^50^ ^,^ ^83^ ^,^ ^84^ Despite these divergent effects, both bile acid classes can activate Nrf2-dependent signaling through the canonical KEAP1-Nrf2 pathway, illustrating the context-dependent nature of Nrf2 activation.^47^ ^,^ ^51^ A similar context dependency is observed also across disease progression. While bile acid-induced stress responses may support epithelial cell survival in pre-malignant or inflamed tissues, certain bile acids, such as lithocholic acid (LCA), have been reported to suppress Nrf2 signaling and enhance oxidative stress-dependent growth inhibition in breast and pancreatic cancer.^48^ ^,^ ^49^

In addition to stress-associated mechanisms, bile acids can exert regulatory effects through activation of nuclear receptors such as the farnesoid X receptor (FXR)^87-89^ and vitamin D receptor (VDR),^90^ which play central roles in bile acid homeostasis and epithelial stress responses. However, current evidence does not support direct transcriptional regulation of Nrf2 by FXR or VDR.

Taken together, current evidence supports Nrf2 as a central mediator of bile acid-induced stress responses, with functional outcomes ranging from cytoprotective adaptation to responses associated with oxidative and genotoxic stress. In contrast, the contribution of upstream bile acid receptors such as FXR and VDR to Nrf2 regulation remains less defined.

### 3.4. Electrophilic and redox-active metabolites

Beyond SCFA and bile acids, gut microbial communities generate electrophilic and redox-active metabolites that impose oxidative or electrophilic stress on host tissues. Mechanistically, these metabolites are closely related to microbiota-derived phenolic compounds (Section 3.5), as both classes converge on Nrf2 signaling through redox-active and electrophilic mechanisms. Certain gut bacteria convert dietary lipids into electrophilic oxo-fatty acids with anti-inflammatory properties. KetoC, generated from linoleic acid by lactic acid bacteria, activates Nrf2 through GPR120-dependent signaling and suppresses NF-κB-driven inflammatory outputs in cell-based and murine models.^66-69^

In addition to oxo-fatty acids, electrophilic aldehydes represent a chemically distinct but conceptually related class of Nrf2 modulators. Metabolites such as acrolein and reuterin-derived electrophiles react with cellular thiols and can be generated through microbial metabolism.^60^ ^,^ ^61^ Electrophilic compounds of this type engage Keap1 electrophile sensing and derepress Nrf2 signaling.^62^ ^,^ ^91^ These reactions induce oxidative and genotoxic stress and may create a selective pressure favoring cells adapted to sustained Nrf2 activation. Accordingly, the biological outcome of electrophilic stress is context-dependent. Consistent with this, the microbial-derived electrophile reuterin has been shown to induce oxidative stress and inhibit colorectal cancer cell growth in association with Nrf2 activation.^63-65^

Extending this redox-modulatory framework, gut microbial gasotransmitters such as hydrogen sulfide and nitric oxide also contribute to epithelial redox balance. Arising from microbial sulfur and nitrogen metabolism, these gases exert concentration-dependent effects on host tissues and have been linked to both epithelial homeostasis and cancer-related processes.^92-95^ Experimental studies in diverse cellular systems suggest that gasotransmitters can activate Nrf2 signaling through Keap1 cysteine modification.^56-58^ However, direct evidence linking microbial gasotransmitter-mediated Nrf2 activation to intestinal tumorigenesis remains limited. Nonetheless, nitric oxide has been shown to activate Nrf2 signaling in colorectal cancer cells, where it can attenuate oxidative stress-induced apoptosis and promote cell survival.^59^ These findings highlight the context-dependent effects of Nrf2 activation.

### 3.5. Polyphenol- and phenolic-derived metabolites

In addition to electrophilic and redox-active metabolites (Section 3.4), microbiota-derived phenolic metabolites arise from bacterial transformation of dietary polyphenols and represent an emerging class of redox-active signals capable of modulating Nrf2-dependent stress responses. Among these, urolithins and the phenolic acid 3,4-dihydroxyphenylacetic acid (DOPAC) provide clear mechanistic links between microbial metabolism, redox regulation and host-tumor interactions.

Urolithins are gut-generated metabolites derived from dietary ellagitannins and ellagic acid, with systemic exposure and inter-individual variability shaped by microbiome composition.^96-99^ Urolithin A (UroA) has been shown to modulate aryl hydrocarbon receptor (AhR) signaling and promote mucus-associated intestinal barrier reinforcement, with links to Nrf2-associated responses.^100^ ^,^ ^101^ UroA has also been shown to engage the Keap1-Nrf2 axis in intestinal epithelial settings relevant to inflammation-associated carcinogenesis. In in vitro and murine colitis models, UroA enhances epithelial barrier integrity and induces canonical Nrf2 target genes, including improved tight junction integrity and reduced epithelial permeability.^39^ In colorectal cancer cell line models, urolithins exhibit tumor-modulatory effects, including induction of autophagy, reduced cell growth, and increased oxidative stress.^41-43^ However, these studies do not directly assess the involvement of Nrf2 signaling.

Another microbiota-derived phenolic metabolite, DOPAC, arises predominantly from microbial metabolism. Fermentation studies identified it as a phenolic metabolite with antiproliferative activity in colon cancer cell models.^102^ ^,^ ^103^ Mechanistically, DOPAC has been shown to activate Nrf2 signaling across multiple experimental settings, including non-cancer contexts, with downstream effects on cellular stress responses such as mitochondrial function and cell survival.^104^ ^,^ ^105^ In addition, Nrf2 activation has been shown to enhance antitumor immune responses, including improved tumor-infiltrating CD8⁺ T-cell fitness and synergy with immune checkpoint blockade.^70^ Other studies in colonic epithelial and cancer cell models report growth suppression and protection against malignant transformation, in association with redox-related mechanisms.^106^ ^,^ ^107^

Collectively, urolithins and DOPAC illustrate how microbiota-derived phenolic metabolites engage Nrf2 across epithelial and immune contexts.

## 3.6. Polyamines

Gut commensal microbes contribute substantially to the intestinal polyamine pool by producing putrescine, spermidine and spermine, which can be taken up by epithelial cells and contribute to the host polyamine pool.^108^ ^,^ ^109^ Through this microbiota-host exchange, polyamines influence epithelial turnover, immune regulation^110-112^ and cellular stress responses.^113^

Polyamines have been linked to Nrf2-dependent stress responses. In particular, spermidine has been shown to activate Nrf2 signaling and enhance antioxidant pathways in models of tissue injury and intestinal inflammation.^52^ ^,^ ^53^ ^,^ ^114^ Conversely, Nrf2 influences polyamine metabolism by regulating key catabolic enzymes such as SSAT, thereby linking redox stress responses to intracellular polyamine turnover.^54^ ^,^ ^115^

Polyamines further shape tumor-immune crosstalk and contribute to immunosuppressive microenvironments.^55^

Although dietary intake can increase systemic polyamine levels in humans, evidence linking polyamines to cancer outcomes remains inconsistent across studies.^116-118^

## 4. Translational strategies targeting the bacterial metabolite-Nrf2 axis

**Figure 3.**
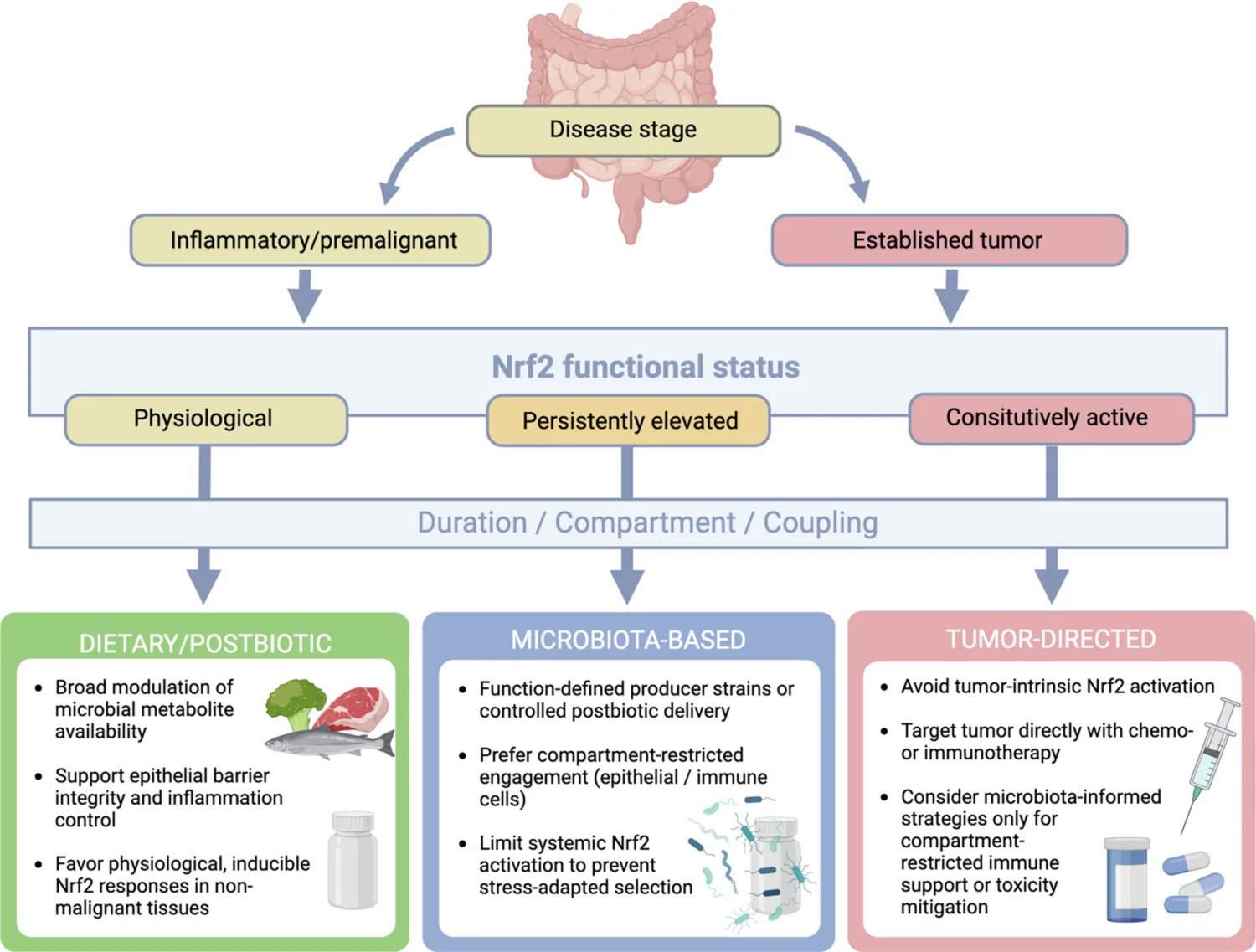
Translational framework for stage- and context-specific targeting of the microbiota-Nrf2 axis. Disease stage influences the functional status of Nrf2, which can range from physiological regulation to stress-induced persistent activation or constitutive activation driven by genomic alterations (e.g., KEAP1/NFE2L2 mutations). The biological effects of Nrf2 depend on activation duration, tissue compartment, and coupling to metabolic and redox stress. In non-malignant or premalignant settings, dietary or postbiotic strategies may support inducible, physiological Nrf2 responses that promote epithelial barrier integrity and immune homeostasis. In conditions of persistent stress-associated activation, microbiota-based approaches using function-defined producer strains or controlled postbiotic delivery may enable compartment-restricted modulation while limiting systemic exposure. In tumors with constitutive Nrf2 activation, further Nrf2 activation in tumor cells should be avoided, and treatment should focus on established anticancer therapies (tumor-directed) such as chemotherapy, radiotherapy or immunotherapy. Microbiota-based strategies may be used to support immune function or reduce treatment-related toxicity rather than to directly target the tumor. Created in BioRender. Pianka, M. (2026) https://BioRender.com/l2cgr2z.

The context-dependent nature of microbiota-driven Nrf2 modulation argues against indiscriminate activation or inhibition strategies. Instead, clinical translation requires stage-dependent, compartment-specific and metabolite-informed approaches that distinguish between protective Nrf2 signaling in healthy tissues and maladaptive Nrf2 engagement in tumor cells. Rather than direct Nrf2 inhibition, more refined strategies include limiting tumor exposure to Nrf2-modulating metabolites, exploiting Nrf2-dependent metabolic rewiring in tumor cells, or selectively targeting downstream Nrf2-dependent programs that sustain tumor fitness. For example, dietary modulation (e.g., fiber intake, polyphenol-rich diets), or microbiota-targeted interventions can influence the availability of Nrf2-modulating metabolites in a disease-dependent manner. Such approaches may be particularly relevant in early tumor stages or inflammatory conditions where microbiota-derived metabolites shape epithelial stress responses. These strategies acknowledge that Nrf2 functions not as a uniformly protective or oncogenic factor, but as a context-sensitive integrator of metabolic and cellular redox state. The following sections translate these concepts into practical frameworks for clinical application (Figure 3).

### 4.1. Molecular stratification and indication selection

Therapeutic modulation of Nrf2 requires molecular stratification. Tumor-intrinsic features, including KEAP1/NFE2L2 mutational status, baseline Nrf2 activity and metabolic configuration, can be used to define clinical contexts in which Nrf2 signaling promotes tumor fitness versus therapeutic vulnerability.^119^ This is particularly relevant given associations between Nrf2 pathway alterations, immune evasion-associated tumor cell phenotypes and reduced responsiveness to immune checkpoint blockade in tumor genotypes characterized by alterations in the KEAP1-Nrf2 pathway.^24^ ^,^ ^120^ In the setting of microbiota-derived metabolite exposure, such stratification becomes essential to distinguish patients in whom Nrf2 engagement is likely to preserve epithelial resilience from those in whom it may reinforce tumor stress adaptation and therapy resistance.

For example, patients with colorectal cancer harboring KEAP1 or NFE2L2 alterations and constitutive Nrf2 activation may be less likely to benefit from interventions aimed at increasing microbiota-derived Nrf2-modulating metabolites, as these pathways are already persistently engaged. In contrast, in patients without baseline Nrf2 hyperactivation, particularly in early-stage disease or inflammatory conditions of the gastrointestinal tract, increasing exposure to barrier-protective microbial metabolites, such as SCFAs or IPA, may support epithelial resilience and potentially reduce inflammation-driven tumor initiation.

### 4.2. Diet, prebiotics and upstream metabolite modulation

Dietary interventions can be implemented to modulate microbial metabolite profiles but require metabolite-based rather than microbiota composition-based endpoints. Microbial community structure alone does not reliably predict tissue-level metabolite exposure, which depends on substrate availability, intestinal transit dynamics and host metabolic configuration. This is exemplified by SCFAs, whose epithelial concentrations and biological effects vary substantially despite similar microbial profiles.^121-123^ While SCFA-induced Nrf2 activation can support epithelial barrier integrity and inflammation control in non-malignant or early disease contexts,^32^ ^,^ ^39^ their effects in established tumors is less predictable and shaped by dose, cellular metabolic state and tumor genotype,^29^ ^,^ ^31^ ^,^ ^32^ ^,^ ^39^ consistent with heterogeneous epidemiological associations observed across colorectal cancer cohorts.^117^

Importantly, dietary and prebiotic interventions lack spatial and cellular specificity and provide limited control over metabolite concentration, exposure duration and cellular target engagement. As a consequence, once Nrf2 signaling is persistently elevated or metabolically rewired, such approaches may fail to achieve selective modulation and could reinforce stress-adapted phenotypes.

Dietary modulation may also redirect microbial tryptophan metabolism toward indole derivatives that support epithelial resilience and immune regulation through AhR-Nrf2-associated pathways. Such strategies are likely most effective in preventing chronic inflammation and maintaining barrier integrity, rather than directly targeting tumor cell-intrinsic stress signaling.^33^ ^,^ ^35^ ^,^ ^36^ ^,^ ^75^ ^,^ ^78^

From a translational perspective, these strategies are therefore most applicable in early-stage disease or inflammatory conditions. For example, in patients with inflammatory bowel disease or individuals at risk of inflammation-driven colorectal cancer, dietary interventions aimed at increasing fiber intake or restoring indole-producing microbiota may enhance epithelial barrier integrity and immune regulation via AhR-associated pathways, indirectly supporting Nrf2-mediated cytoprotective responses and reducing pro-tumorigenic inflammation.

### 4.3. Producer strains, postbiotics and compartment targeting

Function-based selection of microbial producer strains offers a more precise alternative to genus-level probiotic approaches. Rather than relying on taxonomic classification, this strategy can be implemented by selecting or supplementing microbes with defined and validated metabolic outputs, enabling targeted modulation of Nrf2-relevant pathways while minimizing off-target effects. For example, *Clostridium sporogenes* - mediated IPA production, *Lactobacillus reuteri* - derived I3A synthesis, and *Gordonibacter spp.* responsible for urolithin A generation, illustrate how defined microbial metabolic functions can be leveraged to modulate Nrf2-relevant biology.^98^ ^,^ ^124-126^ This approach is particularly relevant for metabolites with pronounced inter-individual variability, such as indole derivatives and urolithins. Although their production depends on substrate availability and overall microbial composition, host exposure often depends on the presence or absence of specific microbial metabolic functions.^75^ ^,^ ^76^ ^,^ ^80^ ^,^ ^96^ ^,^ ^99^ In contrast, SCFA production is supported by multiple, functionally redundant microbial taxa and fermentation pathways.^123^ As a result, SCFA levels are more consistently generated across individuals and are primarily driven by substrate availability and overall microbial metabolic activity. Accordingly, interventions targeting indole or urolithin exposure may require stratification based on microbial metabolic capacity, whereas SCFA-directed strategies are more likely to produce graded, population-wide effects.

From a translational perspective, the application of such strategies requires careful patient stratification. Supplementation with defined producer strains or postbiotics may be most beneficial in patients with impaired microbial metabolic capacity, such as those with dysbiosis, reduced indole-producing taxa, or compromised barrier function in inflammatory conditions of the gastrointestinal tract. In contrast, in patients with established tumors, particularly colorectal cancer characterized by sustained Nrf2 activation, such interventions may require caution, as increased availability of Nrf2-modulating metabolites could reinforce tumor-associated stress adaptation. These considerations highlight the need for compartment-specific strategies that preferentially target non-malignant tissues while limiting unintended activation of tumor cell-intrinsic stress responses.

In addition to selecting the appropriate metabolite and patient group, it is also important to consider where these metabolites exert their effects. Local delivery strategies, such as enteric release or intratumoral administration of defined metabolites, can enhance epithelial or immune effects while limiting systemic Nrf2 activation. Direct postbiotic supplementation further enables controlled exposure and can help overcome variability in microbial metabolite production, as demonstrated for urolithin A in human intervention studies.^40^ ^,^ ^127^

Accordingly, successful clinical translation will depend not only on metabolite selection but also on individualized assessment of microbial metabolic capacity, tissue context, and Nrf2 activation status.

### 4.4. Risk reduction and combination strategies

Translational application of microbiota-Nrf2 biology also includes strategies aimed at de-risking maladaptive metabolic environments. Diet-microbiome configurations enriched in sulfur metabolism have been associated with increased colorectal cancer risk,^11^ ^,^ ^128^ with mechanistic studies implicating microbial sulfur metabolism and hydrogen sulfide production in epithelial dysfunction and inflammatory signaling.^11^ ^,^ ^92^ ^,^ ^93^ ^,^ ^128^ These findings suggest that preventive strategies can be achieved through modifying diet and monitoring relevant metabolic and inflammatory markers, rather than broadly activating stress-response pathways. In such settings, sustained activation of Nrf2, although initially protective, may contribute to maladaptive responses under chronic inflammatory or metabolically unfavorable conditions.

Conversely, disruption of microbial metabolism, for example through antibiotic-induced dysbiosis, can reduce the production of secondary bile acids and impair stress-adaptive signaling pathways, including Nrf2-associated responses, thereby promoting inflammation and epithelial vulnerability. Restoration of microbial metabolic function, for instance through fecal microbiota transplantation (FMT), may re-establish bile acid conversion and associated stress-response signaling in selected clinical contexts.

Beyond their role in prevention, microbiota-derived metabolites also offer opportunities for rational combination strategies. Beyond immune checkpoint blockade, modulation of Nrf2 signaling may influence treatment response by altering tumor cell resistance and host tissue tolerance, as cytotoxic chemotherapy and radiotherapy both rely in part on oxidative and metabolic stress induction. While persistent Nrf2 activation in tumor cells has been linked to chemoresistance^129^ ^,^ ^130^ and radiotherapy resistance^131-133^ in multiple malignancies, controlled activation of Nrf2 in non-malignant tissues may reduce treatment-associated toxicities, including mucosal injury and inflammatory damage.^134-136^ Consistent with this compartment-specific duality, several microbiota-derived metabolites have been shown to enhance immune-cell resilience or improve responses to tumor-directed therapies.^3^ ^,^ ^76^ ^,^ ^80^ ^,^ ^119^

From a clinical perspective, microbiota-informed strategies may be most effectively implemented as adjuncts to established therapies rather than standalone interventions. In patients undergoing chemotherapy or radiotherapy, targeted modulation of microbiota-derived metabolites could be used to protect non-malignant tissues, such as the intestinal epithelium, from treatment-associated oxidative and inflammatory damage. At the same time, these approaches should be designed to avoid systemic or tumor-specific enhancement of Nrf2 signaling, which could contribute to therapy resistance. This may be achieved through temporal control (e.g., peri-treatment interventions), localized delivery, or selection of metabolites with compartment-restricted effects. Such strategies highlight the potential of microbiota-Nrf2 modulation to improve therapeutic tolerability while preserving anti-tumor efficacy.

## 5. Discussion and conclusion

Gut microbiota-derived metabolites represent a host-microbial signaling axis linking microbial metabolism to redox homeostasis, chronic inflammation and cancer.^1^ ^,^ ^2^ However, their biological effects are often inconsistent across experimental and clinical settings, reflecting substantial variability in host and metabolic context. As a result, attempts to assign fixed functional roles to individual metabolites have remained limited in their predictive and translational value. This highlights a critical gap in the field, as current approaches do not adequately explain why the same metabolites can exert divergent biological effects. Here, we propose an integrative framework that links microbiota-derived metabolite signaling to context-specific regulation of cellular stress responses, with Nrf2 as a central integrator, to reconcile these inconsistencies and provide a basis for more coherent translational strategies.

At a mechanistic level, this variability arises from differences in how these metabolites modulate stress response pathways. Rather than acting as uniformly protective or tumor-promoting factors, they fine-tune cellular responses according to tissue compartment, metabolic wiring and disease stage. Across metabolite classes, Nrf2 emerges as a central regulator of these responses, yet its biological consequences diverge between transient inflammation, premalignant adaptation and established malignancy,^3^ ^,^ ^16^ highlighting the need to distinguish between protective and maladaptive Nrf2 signaling across disease stages.

This framework also helps to explain previously reported context-specific effects. For example, SCFAs can reinforce epithelial barrier integrity through Nrf2 activation^39^, but in metabolically rewired tumor cells they may contribute to epigenetic reprogramming and altered stress responses.^30^ ^,^ ^32^ Accordingly, biological outcome appears determined less by metabolite identity than by the metabolic and genetic state of the target tissue, with implications for the timing and context of microbiota-targeted interventions.

While the available evidence consistently points to a central role of Nrf2 in mediating the effects of microbiota-derived metabolites across different biological contexts, direct assessment of Nrf2 signaling remains limited in many studies. As a result, Nrf2 involvement is often inferred rather than experimentally demonstrated, highlighting a gap between conceptual relevance and mechanistic validation and underscoring the need for more systematic interrogation of Nrf2 pathway activity in this context.

The context-dependent effects become particularly evident in premalignant tissues, where sustained Nrf2 engagement may reinforce stress-adapted epithelial populations and narrow the therapeutic window.^3^ ^,^ ^14^ In established tumors, deregulated or mutation-driven Nrf2 activation supports metabolic adaptation and tumor fitness.^17^ High Nrf2 activity has further been linked to resistance to chemotherapy^129^ ^,^ ^130^ and radiotherapy,^131-133^ particularly in KEAP1/NFE2L2-altered tumors.^24^

Beyond tumor-intrinsic effects, Nrf2 signaling also plays a critical role within the tumor immune microenvironment. Emerging evidence indicates that Nrf2 can promote immunosuppressive programs in myeloid cell populations, thereby contributing to tumor progression and resistance to immunotherapy.^20-22^ In contrast, its role in lymphoid compartments appears more nuanced, as redox-sensitive Nrf2 signaling can either impair or enhance T-cell function depending on the activation state and microenvironmental conditions.^23^ In parallel, tumor-intrinsic activation of the KEAP1-Nrf2 pathway has been associated with immune evasion and reduced responsiveness to immune checkpoint blockade.^24^

Nrf2 also contributes to stromal adaptation, promoting tumor-supportive programs in cancer-associated fibroblasts, while limiting tumor progression in non-malignant stromal compartments.^25^ ^,^ ^26^ Collectively, these findings indicate that Nrf2-dependent stress responses in the tumor microenvironment are highly context-dependent and may represent a potential interface for modulation by microbiota-derived metabolites.

These multi-compartment effects have direct translational implications. Broad dietary or microbiome-modifying strategies are unlikely to exert predictable effects in contexts where Nrf2 signaling is persistently elevated or genetically deregulated. While selected metabolites such as reuterin have demonstrated tumor-suppressive effects via redox modulation,^65^ sustained tumor cell-intrinsic Nrf2 activation may conversely buffer oxidative stress induced by chemotherapy or radiotherapy.

At the same time, controlled Nrf2 activation in non-malignant tissues may mitigate therapy-associated toxicity, including radiation-induced tissue damage^134^ ^,^ ^136^ and chemotherapy-associated oxidative injury.^135^ These observations underscore the need for spatially and temporally controlled, compartment-specific modulation strategies, that preserves epithelial or immune resilience without reinforcing tumor-intrinsic stress tolerance.

Future strategies should therefore move beyond taxonomy-driven microbiome modulation toward metabolite-, mechanism- and genotype-informed approaches. Precision-guided manipulation of the microbiota-Nrf2 axis may help limit inflammation-driven tumor initiation and mitigate treatment-related toxicity, provided that intervention timing, tissue targeting and baseline Nrf2 activity are carefully considered. Equally important, future studies should systematically assess Nrf2 pathway activity across different biological contexts to establish causal links between microbiota-derived metabolites and Nrf2-dependent stress responses.

## Supplementary Material

S1_Metabolite_nrf2_review.docx

## Data Availability

No new data were generated or analyzed in this study.
