# Supplementary material for: Gut microbiota-derived metabolites as context-dependent modulators of Nrf2 signaling in inflammation-driven carcinogenesis
Source: Gut Microbes. 2026 Aug 20;18(1):2718561. doi: 10.1080/19490976.2026.2718561 (PMC13502030; doi:10.1080/19490976.2026.2718561)
Supplement: Supplementary file 1 [file KGMI_A_2718561_SM7476.docx]

# S1. Microbiota-derived metabolites modulating Nrf2 signaling in context of carcinogenesis

The table summarizes representative metabolites within each class and their reported effects on Nrf2-related pathways. Effects are highly context-dependent and reflect the experimental systems studied; absence of tumor-promoting effects does not imply tumor-suppressive activity. Metabolites were selected based on their microbiota-derived origin and available evidence for modulation of Nrf2 signaling, either directly or via associated redox and stress-response pathways, in cancer initiation, progression, or therapy response.

| **Metabolite class** | **Specific metabolite** | **Experimental context** | **Direct / indirect / inferred Nrf2 involvement** | **Biological outcome** | **Key reference(s)** |
| --- | --- | --- | --- | --- | --- |
| SCFA | Butyrate | Non-malignant intestinal epithelium; DSS colitis (mouse) | Nrf2 activation (redox, mitophagy) | Barrier protection, anti-inflammatory | 22 |
|  |  | Human CRC cell lines (Warburg phenotype) | Indirect or context-dependent Nrf2 involvement | Growth suppression, epigenetic reprogramming \| | 23, 24, 25 |
| Indoles | Indole-3-propionic acid (IPA) | Intestinal epithelium | Nrf2 activation (barrier genes) | Enhanced epithelial barrier | 37 |
|  |  | Breast cancer cell lines | Oxidative stress but no direct evidence for Nrf2 involvement | Cytostatic effects; growth suppression | 38 |
|  | Indole-3-lactic acid (ILA) | Intestinal epithelium; ischemia/reperfusion | AhR-Nrf2 crosstalk | Barrier and vascular protection | 32, 34 |
|  |  | Colorectal cancer models | Metabolic reprogramming; Nrf2 involvement not established | Tumor growth suppression | 39 |
|  | Indole-3-aldehyde (IAld / I3A) | Immune and epithelial compartments | AhR-associated signaling (Nrf2 involvement not established) | Immune homeostasis; inflammation control | 40 |
| Urolithins | Urolithin A | Intestinal epithelium | Nrf2-activation | Barrier protection | 82 |
|  |  | Human supplementation study | Mitophagy (Nrf2 involvement not established) | Enhanced mitochondrial and cellular health | 117 |
|  |  | CRC cell lines | ROS-associated stress (Nrf2 involvement not established) | Cell cycle arrest; apoptosis | 83-85 |
| Bile acids | Ursodeoxycholic acid | Hepatic and esophageal epithelial cells | Nrf2 activation | Cytoprotection; antioxidant response | 45, 47, 48 |
|  | Lithocholic acid | Cancer vs non-malignant cells | Electrophilic/oxidative stress with Nrf2 activation (bile acid-associated) | Cytotoxic or adaptive responses (context-dependent) | 52, 55, 56 |
|  | Deoxycholic acid (DCA) | Barrett / epithelial cells | Nrf2-mediated stress-induced adaptive response | Protection against oxidative stress and DNA damage; enhanced cell survival | 50, 51 |
| Polyamines | Spermidine | Colitis; liver injury models | Nrf2 activation (non-canonical) | Barrier protection; antifibrotic effects | 100, 101 |
|  |  | Cancer models | Context-dependent stress adaptation (Nrf2 involvement not established) | Therapy resistance (context-dependent) | 103, 104 |
| Gasotransmitters | Hydrogen sulfide (H₂S) | Intestinal and vascular tissues | Keap1 S-sulfhydration → Nrf2 activation | Cytoprotection; adaptive stress response | 72, 73 |
|  | Nitric oxide (NO) | Colon carcinoma and epithelial cells | Keap1 S-nitrosylation → Nrf2 activation | Redox adaptation; survival signaling | 74, 75 |
| Electrophiles | Acrolein | Epithelial cells | Electrophilic Keap1 modification → Nrf2 activation | Redox stress response | 61-63 |
|  | Reuterin | CRC models; epithelial cells | Electrophilic stress (Nrf2 involvement not established) | Redox stress; tumor suppression | 65-67 |
|  | Fatty acid derivatives, 10-oxo-octadecenoic acid (KetoC) | Epithelial and immune cells | Electrophilic stress-mediated Nrf2 activation | Anti-inflammatory; cytoprotection | 57, 58, 60 |
| Phenolics | 3,4-Dihydroxyphenylacetic acid (DOPAC) | CRC models; immune cells | Nrf2-mediated mitophagy | Tumor suppression; immune activation | 90 |
